# DNA microarrays to identify etiological agents, as sensors of environmental wellbeing

**DOI:** 10.3389/fbioe.2023.1085976

**Published:** 2023-04-24

**Authors:** María Leticia Arena-Ortiz, Ernesto Cuauhtemoc Sánchez-Rodríguez, Javier Eduardo Apodaca-Hernández, Joanna María Ortiz-Alcántara, Karen Ríos-Contreras, Xavier Chiappa-Carrara

**Affiliations:** ^1^ Ecogenomics Laboratory, National Autonomous University of Mexico (UNAM), Yucatan, Mexico; ^2^ Hyperbaric Medicine Department at Hospital Agustín O’Horan, Health Ministry of Yucatán, Postgraduate Department of Faculty of Medicine of National Autonomous University of Mexico (UNAM), and Global Health Institute, Michigan State University (MSU), Mérida, Mexico; ^3^ Bibliotecas Genómicas S.A, Merida, Mexico; ^4^ Conservation Biology Laboratory, National Autonomous University of Mexico (UNAM), Yucatan, Mexico

**Keywords:** biosensor, etiological agents, environmental wellbeing, public health, dnamicroarray

## Abstract

**Background:** The epidemiologic transition in Mexico has generated a change of paradigm in public health. Morbidity is characterized by infectious diseases and the mortality is due to chronic degenerative diseases. The three most important infectious diseases in the country are: respiratory infections, diarrhea, and urinary tract infections.

**Method:** The objective of this work was to build a tool to monitor the presence of health risks in the environment in a timely manner and to demonstrate its application in different sicknesses, especially those that are water related. In this study, we analyzed water samples from five cenotes with high tourist flow in the State of Yucatan. We developed a DNA microarray for the adequate and prompt detection of viruses, bacteria, fungi, and parasites. This microarray could be used in samples of different origin including air, water (fresh, brackish and saltwater), food, inert surfaces or wounds. Clinically, it would allow prompt and precise detection of etiological agents of infectious diseases to prevent outbreaks. It would also be useful for the identification of those agents that cannot be detected in our laboratories with the traditional methods. It includes 38,000 probes that detect 252 etiological agents of diseases in humans and antimicrobial resistance genes. Results from DNA samples can be obtained in 24 h, which would be difficult or impossible using other technologies.

**Results:** The results are readily available within 24 h. Samples from five cenotes (sinkholes) with high flow of people, were analyzed with the microarray. The water samples analyzed detected 228 different bacteria, viruses, fungi, and protozoa. They are amongst the most important etiological agents for infectious diseases in Mexico.

**Conclusions:** The microarray provides the opportunity for precise and early detection of various infectious agents in individuals, hospitals and natural environments. This could help reduce the global burden of diseases, the severity of outbreaks, and reduce antibiotic resistance.

## 1 Introduction

There is a great need to address the growing impact of the environmental conditions on public health. Some examples are environmental pathogens that cause respiratory infections, diarrhea, hospital infections, and bacterial resistance to antibiotics.

Using information from a data bank of diseases that are monitored monthly in health institutions in Mexico, we developed a DNA biosensor for the early and prompt detection of these pathogens in air, water (fresh, salt, and brackish), food, inert surfaces, and also in wounds. This could become a key monitoring device for health institutions, in case of outbreaks and other infectious risks.

Microarray technology was developed to analyze thousands of genetic sequences at once and is used for its sensibility and for the great amount of information that can be obtained from a single chip in short times, using laboratory resources more efficiently. Results can be obtained in 24 h from DNA sample collection, without the need of cultivating or amplifying the targets, task that would be difficult or impossible using other technologies. Probes of DNA or RNA are attached to a solid surface. Then, the sample of genetic material is hybridized with the probes in the device and the pairing of complementary bases can be detected using fluorescence that is quantified by the reader, allowing the identification of the target genes (Boussioutas and Haviv, 2003; Dudda‐Subramanya et al., 2003).

Our biosensor called “Yucateco” contains 38,000 probes that are species-specific for a set of organisms that were selected due to their public health importance, since they directly cause multiple infections. It allowed the detection of 1 arthropod, 112 bacteria, 29 fungi and yeasts, 31 microalgae, 8 nematodes, 8 flatworms, 11 protozoa, 40 viruses that cause diseases in humans and 12 antimicrobial resistance genes to betalactam agents, fluroquinolones, newer generation of cephalosporins and carbapenems genes (Supplementary Table S1). Eighty-five percent of them are identified to the species level and the rest to the level of genus. In the case of viruses and bacteria where all the species included in the same genus are considered pathogenic, it would be enough to detect potential risks to human health identifying organisms to the taxonomic level of genus.

The design and specificity of the probes was validated through bioinformatic challenges by our team and by Affymetrix, now part of Thermo Fisher Scientific that manufactured the array for the GeneChip^®^ system. Furthermore, the microarray was tested using a control sample prepared with bacterial DNA in different concentrations to simulate the behavior of environmental samples.

The objective of this work was to use this biosensor to evaluate the water quality in five sinkholes (cenotes) of the Yucatan Peninsula. They have a vital importance as a water source but could also become a reservoir of infectious diseases. The Yucatan peninsula holds 85% of the underground water reserve of the country and cenotes are not only part of it but they have great cultural, religious, ecological, and touristic importance. Anthropogenic contaminants in water are a major issue in the area. Human contact with contaminated water for recreational use in natural environments could become a public health issue (Hoogesteijn-Reul et al., 2015; Aguilar-Duarte et al., 2016; Batllori and Chávez-Guzmán, 2016; Chávez-Guzmán, 2016; Hernández Flores, 2018).

## 2 Materials and methods

### 2.1 Sample collection

**FIGURE 1:**
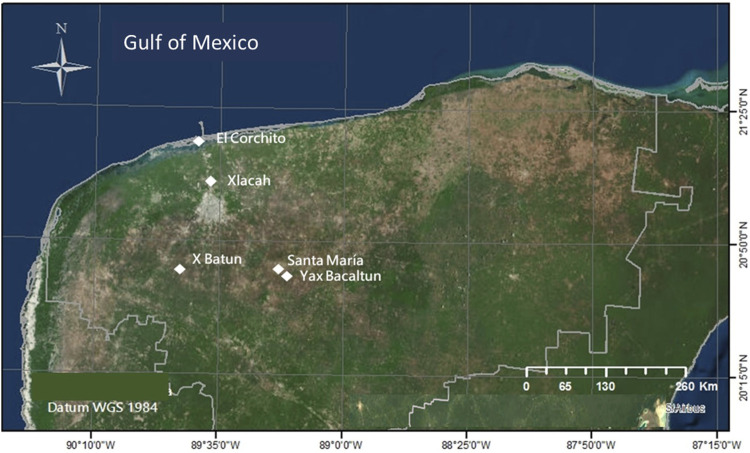
Map of the Yucatan peninsula with the location of sampled cenotes including Pájaros (Reserva Ecológica El Corchito), Xlacah (Zona Arqueológica de Dzibilchaltún), X΄batun (San Antonio Mulix), Yaxbacaltun and Santa María (Homún).

Water samples were collected from five cenotes with high tourist activity in the State of Yucatan, Mexico, including: Pájaros (in the natural protected area of El Corchito), Xlacah (in the Archeological zone of Dzibilchaltún), X΄batun (San Antonio Mulix municipality), and Yaxbacaltun and Santa María (Homún municipality) (Figure 1). Physicochemical parameters of the water were measured *in situ*, with a multiparameter meter (YSI, OH, United States). These included: temperature, pH, salinity, conductivity, dissolved oxygen, total dissolved solids, and redox potential. Water samples were collected in polyethylene containers of 4 L in triplicates and transported in an ice cooler to the Ecogenomics laboratory to be stored at 4°C for up to 24 h. Four liters of water were filtered on a sterile nitrocellulose membrane of 0.45 μm pore size (Millipore, Darmstadt, Germany). The filters were stored at −20°C until DNA extraction.

### 2.2 DNA extraction

Metagenomic DNA was extracted from the filters according to a silica-based protocol previously reported (Rojas-Herrera et al., 2008). The DNA integrity was checked on a 1% agarose gel. dsDNA concentration and purity were determined using the QuantiFluor^®^ dsDNA kit in a Quantus Fluorometer (Promega Corporation, Madison, WI, United States) and the Multiskan Go Spectrophotometer (Thermo Scientific Waltham, MA, United States), respectively, following the manufacturer’s protocols. The DNA obtained from the filters of each sampling site was pulled to be analyzed in a single chip. The total amount of DNA used as input for each array was 3.01 ng from X΄batun, 178.8 ng from Santa María and Yaxbacaltun, 269.5 ng from Xlacah, and 490 ng from Pájaros cenote.

### 2.3 Control sample preparation

To simulate the behavior of environmental samples where a complex mixture of genomes is present, a control sample was prepared including 1 µL of DNA of each of the following species, in different quantities: *Acinetobacter baumannii*, 22 ng; *Bordetella pertussis*, 0.27 ng; *Bordetella parapertussis*, 18 ng; *Enterobacter cloacae*, 46 ng; *Escherichia coli* (enteropathogenic), 191 ng; enteroaggregative, 166 ng; enteroinvasive, 95 ng; enterotoxigenic, 200 ng; diffusely-adherent, 154 ng; adherent-invasive, 160 ng; *Helicobacter pylori*, 60 ng; *Klebsiella pneumoniae*, 125.0 ng; *Klebsiella oxytoca*, 28.0 ng; *Mycobacterium tuberculosis*, 7.5 ng; *Mycobacterium avium*, 1.53 ng; *Mycobacterium kansasii*, 1.25 ng; *Neisseria meningitidis*, 0.09 ng; *Pseudomonas aeruginosa*, 48 ng; *Shigella* sp., 27 ng; *Staphylococcus aureus*, 2.6 ng; *Vibrio cholerae*, 1.44 ng; *Vibrio parahaemolyticus*, 4.83 ng; antimicrobial resistance genes (QniA, 23 ng; Qni B, 9 ng; VIM, 24 ng; TLA, 33 ng; IMP, 30 ng; SHV, 14 ng; NDM, 31 ng; CTXM, 25 ng; GES, 19 ng; aac, 19 ng).

### 2.4 DNA microarrays

A customized microarray was designed containing 38,000 specific probes (patent application MX/a/2018/014650) to detect 269 pathogens and antimicrobial resistance genes in a single chip. The chip was manufactured for the GeneChip^®^ platform by Affymetrix (Applied Biosystems Microarrays, Thermo Fisher Scientific, Waltham, MA, United States). The metagenomic DNA was processed following a protocol optimized for a complex mixture of genetic materials, using the Affymetrix^®^ SNP 6.0 Core Reagent Kit (Thermo Fisher Scientific, Waltham, MA, United States). Briefly, end-labeled fragments of DNA were generated with DNase I, biotin-labeled nucleotides and a deoxy terminal transferase (TdT). Labeled DNA was hybridized to the chip at 49 °C for 16 h in a GeneChip^®^ 645 oven (Thermo Fisher Scientific, Waltham, MA, United States), then washed and dyed in the GeneChip^®^ Fluidics Station 450 (Thermo Fisher Scientific, Waltham, MA, United States), and scanned in a GeneChip^®^ Scanner 3,000 7G (Thermo Fisher Scientific, Waltham, MA, United States). The etiological agents in each sample were determined according to the pixel intensity data in each cell at the specific probe locations in the microarray.

### 2.5 Data analysis

The analysis of the CDF files generated after scanning the chip was performed with a pipeline using the Bioconductor package (https://www.bioconductor.org/) and R programming language (https://cran.r-project.org/). Specific libraries include *makecdfenv* to build the work environment; *affxparser* to delimit data from plain text and define working columns; and *affy* to manage specific data from Affymetrix. The values referring to the grid were customized for this “Yucateco” microarray. From the raw data file, the pixel intensity values were obtained, the values from a blank sample not containing DNA were subtracted and considered a negative control. Additionally, by convention, a cut-off point was determined for the assignment of a positive sample, thus all those values below 30% of pixel intensity were considered as negative. A list of etiological agents was created based on this criterion for each sample. Subsequently, using the *batch blast entrez* algorithm (https://www.ncbi.nlm.nih.gov/sites/batchentrez) the complete genetic annotation was retrieved from the databases corresponding to the access numbers of each pathogen, to assign the names including genus, species and, in some cases, genotype. The etiological agents were classified according to the systems that they can affect in the following categories: gastrointestinal, respiratory, nervous system, muscle and soft tissue, immunological, ophthalmological, cardiovascular, urinary and other, including otic, congenital and bones. A cluster analysis constructed using the Euclidean distance as a measure of association, allowed to obtain a dendrogram in which a cut-off value equal to 5 was used to determine the degree of dissimilarity between the sites sampled with relation to the presence or absence of the targeted species was carried out with the Primer 6 software (Villardón, 2007).

## 3 Results

The DNA concentration obtained from environmental samples is directly related to several factors, therefore it is not feasible to set a constant number of liters of water required per analysis. A control sample was prepared to mock an environmental sample, where a complex mixture of genomes can be found, including purified DNA from 22 bacterial species and 10 antimicrobial resistance genes. The amount of DNA spiked in the control sample ranged from 0.09 to 200 ng, corresponding to *Neisseria meningitidis* and enterotoxigenic *E. coli,* respectively, both being detected by the array. A total of 18 out of 22 species were detected by the microarray. However, some species such as *Mycobacterium kansassi* (1.25 ng), *M. avium* (1.53 ng)*, S. aureus* (2.6 ng) and *Acinetobacter baumanii* (22 ng) were not detected by the array in the control sample. In the case of resistance genes, 6 out of 10 were detected, the amount of DNA used as input ranged from 14 to 31 ng. In this study, it was necessary to filter up to 12 L of water from each cenote to obtain at least 0.1 ng/μL of DNA to be able to have reading results in the array. The total DNA used as input for each array was in the range of 3.01 ng from X΄batun to 490 ng from Pájaros cenote, while 178.8 ng from Santa María and Yaxbacaltun, and 269.5 ng of DNA from Xlacah.

**FIGURE 2:**
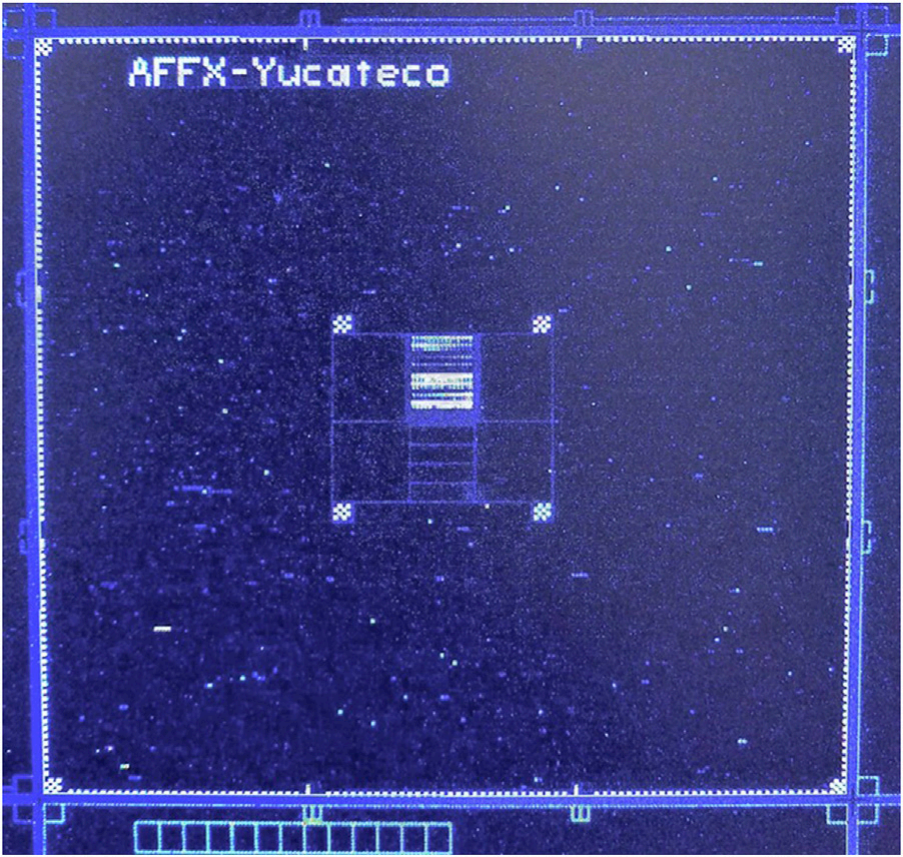
Image of the Yucateco array hybridized with the water sample from the Pájaros cenote in Reserva Ecológica El Corchito. Pixels where DNA fragments hybridized at the specific probes in the microarray appear in blue and hybridization controls in white.

The presence or absence of etiological agents in the samples were determined according to the pixel intensity in each area of the array defined as a cell. Detection of the fluorescence emitted by the DNA fragments hybridized at the specific probes in the microarray, resulted in an image of points called pixels, with coordinates and values of fluorescence intensity (Figure 2).

**FIGURE 3:**
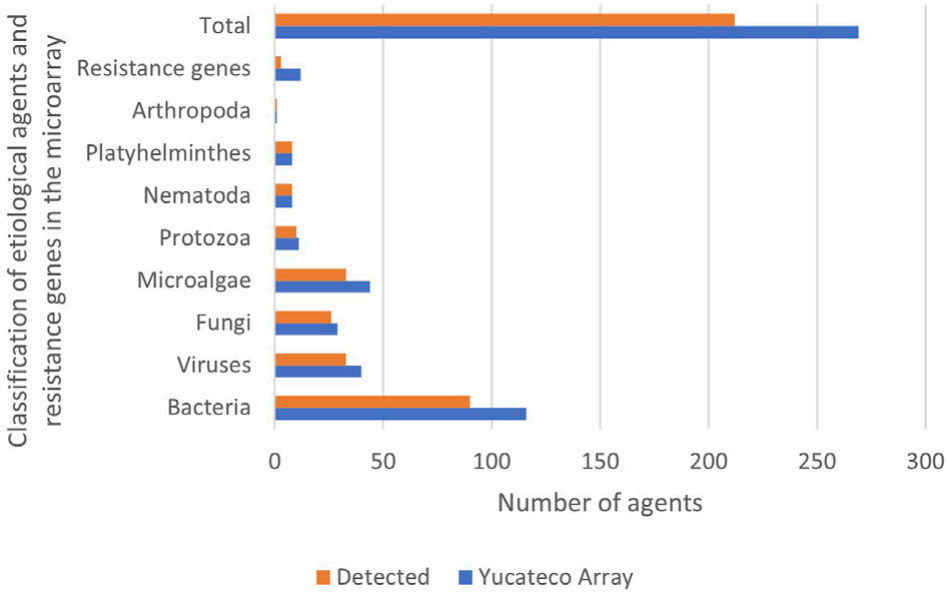
Etiological agents and resistence genes included in the microarray and detected in cenotes by classification.

The etiological agents found in the water samples included pathogens that represent a threat to humans as well as other microorganisms that are not harmful but could cause diseases to other species. Some species of microalgae and other microorganisms were included in the microarray due to their potential biotechnological use, and not because they represent a health risk. With the use of the “Yucateco” array a total of 209 agents were detected from the 269 targeted, including 90 bacteria, 33 viruses, 26 fungi and yeasts, 33 microalgae, 10 protozoa, 8 nematodes, 8 platyhelminthes, 1 arthropod, and some resistance genes (Figure 3).

**FIGURE 4:**
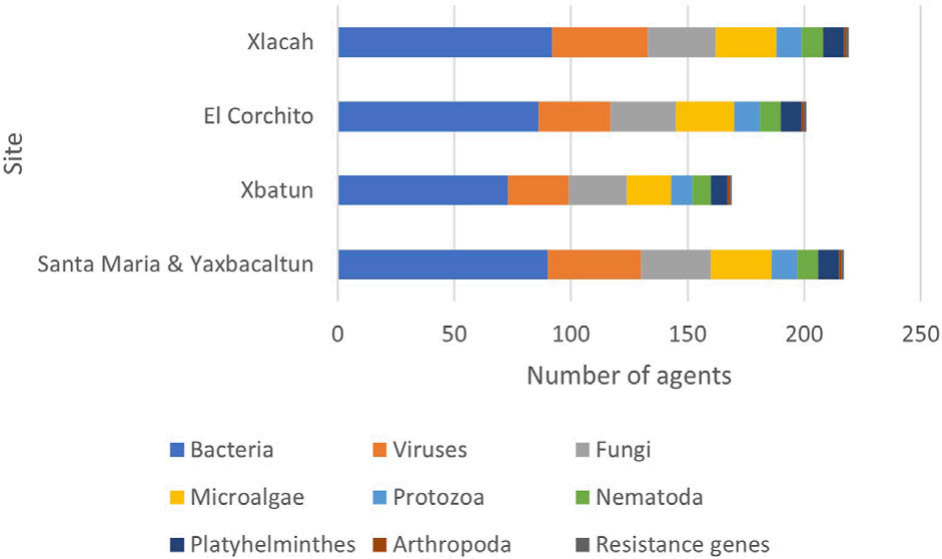
Etiological agents detected by cenote. The number of agents detected in each cenote according to its classification are presented, a total of 228 agents were detected in the samples.

In some cases, more than one species was detected with a total of 228 species found in the samples. The number of species detected in each cenote according to its classification are presented in Figure 4. Results show that Xlacah is the cenote where the highest number of species were identified, with 218 species. Both, Santa Maria and Yaxbacaltun cenotes presented a total of 216 species, followed by the Pájaros cenote, with 200 species. In the X΄batun cenote, 168 species of human pathogens were found.

**FIGURE 5:**
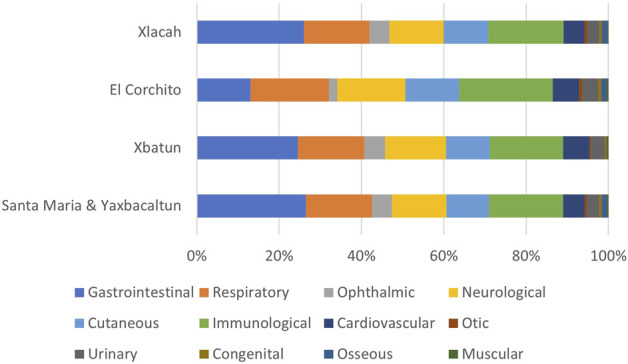
Human systems affected by diseases caused by etiological agents detected in cenotes. Etiological agents were classified according to the systems that can affect in the following categories: gastrointestinal, respiratory, nervous system, muscle and soft tissue, immunological, ophthalmological, cardiovascular, urinary and other, including otic, congenital and bones.

The diseases associated with the pathogens detected in the cenotes were classified according to the affected system (Figure 5). Gastrointestinal and respiratory etiological agents were the most commonly identified species, while species that affect the muscular and osseous systems, were the least common. It is important to consider that a single etiological agent can affect more than one system at the same time.

**FIGURE 6:**
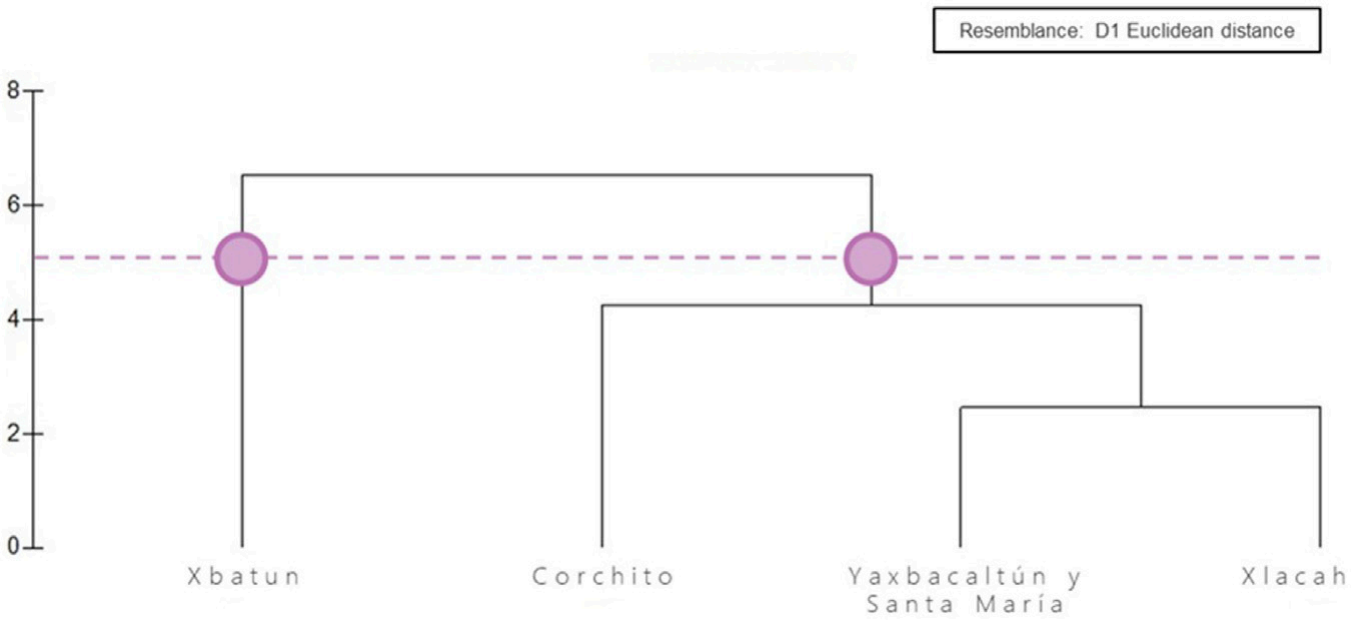
Cluster analysis. A cut-off value of 5 was used to determine the degree of dissimilarity between the sites with relation to the presence or absence of the detected species. The Euclidean distance was chosen as a measure of association..

The cluster analysis allowed cenotes to be assembled in two groups (Figure 6). The first was formed only by the X΄batun cenote, and the second group which includes similar cenotes based on the species detected is formed by Xlacah, Santa Maria and Yaxbacaltun, and El Pájaro. Xlacah, and Santa Maria and Yaxbacaltun cenotes formed a subgroup within the second cluster.

## 4 Discussion

It is considered that in developing countries around 70% of the diseases are transmitted by water. In Yucatán, the three most common infectious diseases are respiratory, gastrointestinal, and urinary, reaching 88.88% of reported infections (Servicios de Salud de Yucatán Dirección General de Epidemiología, 2021). In these countries, gastroenteritis is responsible for many deaths in the first year of age. Later in life, there is a predisposition to acquire respiratory disease that compromise the immune system, already affected by malnourishment. Detection of all the etiological agents is difficult since it is presumed that we can only culture between 10% and 15% of all the infectious pathogens reported by health services. The early identification of these pathogens could improve the results of the treatment and reduce the resistance generated by these agents.

Microarrays are a sensitive tool that can be used for quick detection of viruses and bacteria from complex clinical samples, that are key for public and veterinary health, as well as food safety (Gardner et al., 2010; Thissen et al., 2019). The “Yucateco” microarray is a biosensor designed to detect pathogens of indirect transmission reported in the epidemiological surveillance conducted yearly in the Yucatán peninsula by the Ministry of Health. Early *in situ* detection of these pathogens with the “Yucateco'' array could be a useful tool for governments to prevent epidemiological outbreaks. The detectable etiological agents include many microorganisms that are not only found in Yucatán. The array also contains probes for detecting other agents not yet reported in the region, which could even represent health risks globally, such as Ebola virus. Therefore, the “Yucateco” microarray can be used to analyze samples worldwide, in any laboratory where the GeneChip^®^ system is available. It could be helpful to identify local or global health risks, from different samples, including soil, water, food, inert surfaces, and wounds. The input sample analyzed by the “Yucateco” array was DNA purified directly from environmental samples without the need of cultivating the microorganisms. The GeneChip^®^ high-density microarrays as the Yucateco offer several advantages. They include multiple 25-mer probe sets of each target, allowing multiple independent measurements, for an accurate evaluation that balances sensitivity and specificity. They also provide high resolution scans and simplified sample preparation, then the results are obtained using time and resources more efficiently than with other technologies. Most of the pathogens included in the probes of the device were detected in cenotes. However, some microorganisms were not found. The genus *Vibrio* contains 66 species, 12 are pathogenic (Franco-Monsreal et al., 2014), 10 of them are included in the microarray but 5 were not detected. For example, *Vibrio fluvialis* is more abundant in the marine environment and was not found in sweet water from the cenotes. Another marine species that was not detected by the array was *Prorocentrum minimum* (Saburova and Chomérat, 2016). Other microorganisms not detected are scarce in nature, such as *Tsukamurella* mostly reported in soil and mud samples (Bratcher, 2018).

Etiological agents of zoonotic diseases were detected, including *Coxiella burnetti,* bacteria of the genera *Leptospira*, *Ehrlichia*, *Borrelia*, *Rickettsia*, viruses such as Hantavirus, rabies virus and Influenza, and the protozoa *Trypanosoma cruzi.* This could be expected since many wild animals such as rodents and bats, are reservoirs of zoonotic diseases in the Yucatán peninsula (Reyes-Novelo and Ruiz-Piña, 2011).

Gastrointestinal pathogens which are amongst the most abundant fecal bacteria, having humans as the sole reservoir and transmitted by the ingestion of contaminated water, including *Shigella sonnei* and *dysenteriae*, and *V. cholerae* (World Health Organization, 2005; Azman et al., 2013), were detected in all cenotes studied.

Additionally, our biosensor has detected some resistance genes in samples of the localities, Corchito (*Raoultella planticola* strain RP01 plasmid pRP01 metallo-beta-lactamase blaNDM-1 gene), Santa María (*K. pneumoniae* strain 1121740 class B beta-lactamase blaNDM-16 gene), Xbatún (*Proteus mirabilis* strain AMP_C_M_3829_10 AmpC bla gene), and Xlacah (*A. baumannii* carbapenem-hydrolyzing beta-lactamase OXA-58 bla-oxa-58 gene). This is crucial information for the implementation of sanitation programs. Therefore, the microarray also could be applied for monitoring hospital environments.

Data generated using the “Yucateco” array can be used to better understand the health status of the ecosystems and the impact of anthropogenic disturbances in the environment. Other technologies to detect etiological agents, such as microbiology techniques, or those derived from molecular biology, such as PCR, have been widely used. However, in all the alternatives it is necessary to carry out an analysis for each of the pathogen species. The innovative element of this biosensor is its capacity to detect up to 269 pathogens in a single experiment. This translates into an efficient use of financial and human resources, and particularly shortening detection times. Early detection can make the difference between an epidemiological outbreak or that it does not occur by applying the appropriate control measures.

## Data Availability

The raw data supporting the conclusion of this article will be made available by the authors, without undue reservation.
