# Supplementary material for: DNA microarrays to identify etiological agents, as sensors of environmental wellbeing
Source: Front Bioeng Biotechnol. 2023 Apr 24;11:1085976. doi: 10.3389/fbioe.2023.1085976 (PMC10165067; doi:10.3389/fbioe.2023.1085976)
Supplement: Supplementary file 1 [file Table1.docx]

|  | **Biological agent** | **Disease** |
| --- | --- | --- |
| **Animalia; Arthropoda** | *Sarcoptes scabiei* | Scabies |
| **Bacteria** | *Acinetobacter baumannii* | Bacteremia, urinary tract infection, wound infection, pneumonia, meningitis, endocarditis |
|  | *Acinetobacter calcoaceticus* | Bacteremia, pneumonia |
|  | *Aeromonas caviae* | Gastroenteritis, skin and soft tissue infection, bacteremia |
|  | *Aeromonas dhakensis* | Gastroenteritis, skin and soft tissue infection, bacteremia |
|  | *Aeromonas hydrophila* | Gastroenteritis, skin and soft tissue infection, bacteremia |
|  | *Aeromonas jandaei* | Gastroenteritis, skin and soft tissue infection, bacteremia |
|  | *Aeromonas schubertii* | Gastroenteritis, skin and soft tissue infection, bacteremia |
|  | *Aeromonas veronii* | Gastroenteritis, skin and soft tissue infection, bacteremia |
|  | *Alcaligenes sp.* | Bacteremia and sepsis |
|  | *Anabaena sp.* | Neurotoxin and hepatotoxin poisoning |
|  | *Aphanizomenon sp.* | Neurotoxic poisoning |
|  | *Arthrobacter oxydans* | Wound, ocular, bacteremia and lung biopsy infection |
|  | *Bacillus anthracis* | Anthrax |
|  | *Bacillus cereus* | Gastroenteritis |
|  | *Bordetella parapertusis* | Whooping cough |
|  | *Bordetella pertussis* | Whooping cough |
|  | *Borrelia burgdorferi* | Lyme's desease |
|  | *Borrelia mazzottii* | Relapsing fever |
|  | *Borrelia recurrentis* | Relapsing fever |
|  | *Brucella melitensis* | Brucellosis, meningitis |
|  | *Burkholderia cepacia* | Bacteremia, respiratory infection, urinary infection, arthritis, peritonitis |
|  | *Burkholderia mallei* | Glanders |
|  | *Burkholderia pseudomallei* | Melioidosis |
|  | *Campylobacter coli* | Gastroenteritis, bacteremia |
|  | *Campylobacter fetus* | Gastroenteritis, bacteremia |
|  | *Campylobacter jejuni* | Gastroenteritis, bacteremia, meningitis |
|  | *Campylobacter laridis* | Gastroenteritis |
|  | *Cardiobacterium spp.* | Endocarditis |
|  | *Cedecea lapagei* | Pneumonia, peritonitis, bacteremia, urinary tract infection |
|  | *Chlamydia psittaci* | Pneumonia |
|  | *Chlamydia trachomatis* | Respiratory psittacosis |
|  | *Chlamydophila pneumoniae* | Ocular trachoma |
|  | *Clostridium botulinum* | Botulism |
|  | *Clostridium difficile* | Gastroenteritis |
|  | *Clostridium perfringens* | Gastroenteritis, enterocolitis, myonecrosis |
|  | *Clostridium tetani* | Tetanus |
|  | *Corynebacterium afermentans* | Endocarditis, brain and kidney abscesses |
|  | *Corynebacterium diphteriae* | Diphtheria |
|  | *Coxiella burnetii* | Q fever, pneumonia, hepatitis, endocarditis, pericarditis, myocarditis, meningoencephalitis, Guillain-Barré syndrome, Miller-Fisher syndrome |
|  | *Ehrlichia canis* | Ehrlichiosis |
|  | *Ehrlichia spp.* | Human monocytic ehrlichiosis |
|  | *Elizabethkingia meningoseptica* | Meningoencephalitis |
|  | *Enterobacter cloacae* | Endocarditis, urinary tract infections, meningitis and intra-abdominal infections |
|  | *Enterococcus faecalis* | Gastroenteritis, hemorrhagic colitis, hemolytic uremic syndrome |
|  | *Escherichia coli (ECEH, ECET,ECEP,ECEI,ECEA,ECAD)* | Gastroenteritis, hemorrhagic colitis, hemolytic uremic syndrome |
|  | *Escherichia coli O157:H7* | Osteomyelitis, septic arthritis, pneumonia, keratoconjunctivitis, bacteremia, peritonitis, sepsis |
|  | *Ewingella americana* | Meningoencephalitis |
|  | *Francisella tularensis* | Tularemia |
|  | *Grimontia hollisae* | Gastroenteritis, bacteremia |
|  | *Haemophilus influenzae* | Pneumonia, meningitis |
|  | *Haemophilus parainfluenzae* | Pneumonia and endocarditis |
|  | *Helicobacter pylori* | Gastritis, gastric carcinoma |
|  | *Klebsiella oxytoca* | Gastroenteritis |
|  | *Klebsiella pneumoniae* | Pneumonia, urinary tract infection, bacteremia, liver abscess |
|  | *Klebsiella variicola* | Bacteremia, meningitis |
|  | *Kocuria rosea* | Acute bacteremia and cholecystitis |
|  | *Leclercia adecarboxylata* | Arthritis, peritonitis, bacteremia |
|  | *Legionella pneumophila* | Legionnaire's disease |
|  | *Leptospira alexanderi* | Leptospirosis |
|  | *Leptospira borgpetersenii* | Leptospirosis |
|  | *Leptospira interrogans* | Leptospirosis |
|  | *Leptospira kirschneri* | Leptospirosis |
|  | *Leptospira noguchii* | Leptospirosis |
|  | *Leptospira santarosai* | Leptospirosis |
|  | *Leptospira weilii* | Leptospirosis |
|  | *Listeria monocytogenes* | Listeriosis, miscarriage |
|  | *Mannheimia haemolytica* | Infection in soft tissues, septic embolization |
|  | *Microcystis aeruginosa* | Poisoning by neurotoxins and hepatotoxins |
|  | *Miscellaneous enterics* | Diarrhea |
|  | *Moraxella catarrhalis* | Otitis, sinusitis, respiratory infections |
|  | *Moraxella lacunata* | Conjunctivitis |
|  | *Mycobacterium avium* | Pneumonia |
|  | *Mycobacterium chelonae* | Pneumonia, skin and soft tissues infection |
|  | *Mycobacterium kansasii* | Chronic respiratory infections |
|  | *Mycobacterium tuberculosis* | Tuberculosis |
|  | *Mycobacterium xenopi* | Pneumonia |
|  | *Neisseria meningitidis* | Invasive meningococcal disease |
|  | *Nocardia asteroides* | Respiratory nocardiosis |
|  | *Ochrobactrum anthropi* | Bacteremia |
|  | *Pantoea agglomerans* | Bacteremia and sepsis |
|  | *Pasteurella sp.* | Hemorrhagic septicemia, atrophic rhinitis, lesions in the respiratory tract |
|  | *Peptococcus sp.* | Pneumonia, otitis, sinusitis |
|  | *Photobacterium damselae* | Necrotizing fasciitis, gastroenteritis, urinary tract infection |
|  | *Plesiomonas shigelloides* | Gastroenteritis, bacteremia, CNS disease, eye infection |
|  | *Proteus mirabilis* | Pneumonia, urinary infections |
|  | *Pseudomonas aeruginosa* | Pneumonia, respiratory, urinary and soft tissue infections, gastroenteritis, bacteremia, joint disorders |
|  | *Rahnella aquatilis* | Bacteremia |
|  | *Raoultella planticola* | Gastroenteritis, respiratory infections |
|  | *Rickettsia spp.* | Rickettsiosis, typhus, fever |
|  | *Salmonella enterica serovar Paratyphi* | Paratyphus fever |
|  | *Salmonella enterica serovar Typhi* | Paratyphus fever |
|  | *Serratia marcescens* | Urinary and respiratory tract infections |
|  | *Shigella sp* | Gastroenteritis |
|  | *Shigella spp.* | Gastroenteritis |
|  | *Staphylococcus aureus* | Gastroenteritis, abscesses, bacteremia, endocarditis, osteomyelitis, pneumonia |
|  | *Staphylococcus haemolyticus* | Bacteremia |
|  | *Stenotrophomonas maltophilia* | Urinary tract infections and bacteremia |
|  | *Streptococcus pneumoniae* | Pneumonia and meningitis |
|  | *Trichodesmium erythraeum* | Respiratory syndrome |
|  | *Tsukamurella spp.* | Pneumonia, skin infections, meningitis |
|  | *Vibrio alginolyticus* | Gastroenteritis, bacteremia, conjunctivitis, peritonitis, abscesses, external otitis |
|  | *Vibrio cholerae* | Cholera |
|  | *Vibrio cincinnatiensis* | Bacteremia, meningitis |
|  | *Vibrio fluvialis* | Gastroenteritis, bacteremia, biliary tract infection, cholangitis, peritonitis, otitis, endophthalmitis, meningitis |
|  | *Vibrio furnissi* | Gastroenteritis |
|  | *Vibrio harveyi* | Bacteremia, wound infection |
|  | *Vibrio mimicus* | Gastroenteritis |
|  | *Vibrio parahaemolyticus* | Gastroenteritis |
|  | *Vibrio splendidus* | Gastroenteritis |
|  | *Vibrio vulnificus* | Gastroenteritis, bacteremia, wound infection |
|  | *Yersinia enterocolitica* | Gastroenteritis, glomerulonephritis, arthritis, myocarditis |
|  | *Yersinia pseudotuberculosis* | Gastroenteritis, glomerulonephritis, arthritis, myocarditis |
| **Fungi** | *Ajellomyces capsulatus* | Acute lung infections |
|  | *Ajellomyces dermatitidis* | Blastomycosis |
|  | *Alternaria spp.* | Allergic rhinitis or hypersensitivity reactions, phaeohyphomycosis |
|  | *Aspergillus flavus* | Aspergillosis |
|  | *Aspergillus niger* | Aspergillosis |
|  | *Aspergillus sp.* | Aspergillosis |
|  | *Aureobasidium pullulans* | Pneumonitis |
|  | *Boeremia exigua* | Keratitis and subcutaneous infections |
|  | *Candida glabrata* | Yeast infection |
|  | *Candida kefyr* | Yeast infection |
|  | *Coccidioides immitis* | Valley fever |
|  | *Coccidioides posadasii* | Valley fever |
|  | *Colletotrichum spp.* | Keratitis and subcutaneous infections |
|  | *Encephalitozoon sp.* | Diarrhea |
|  | *Enterocytozoon bieneusi* | Diarrhea, wasting syndrome, rhinitis, bronchitis, sinusitis, cholangiopathy, acalculous cholangitis, cholecystitis |
|  | *Fusarium graminearum* | Keratitis and subcutaneous infections |
|  | *Fusarium oxysporum* | Keratitis and subcutaneous infections |
|  | *Lacazia loboi* | Lobomycosis |
|  | *Nosema spp.* | Microsporidiosis |
|  | *Paracoccidioides brasiliensis* | Paracoccidioidomycosis |
|  | *Penicillium digitatum* | Allergic responses, hypersensitivity pneumonitis, and asthma |
|  | *Penicillium italicum* | Allergic responses, hypersensitivity pneumonitis, and asthma |
|  | *Pichia kudriavzevii* | Asthma |
|  | *Pleistophora sp.* | Fungaemia, endophthalmitis, arthritis and endocarditis |
|  | *Pneumocystis jirovecii* | Myositis |
|  | *Saccharomyces cerevisiae* | Respiratory tract infections |
|  | *Talaromyces variabilis* | Yeast infection |
|  | *Trachipleistophora hominis* | Diarrhea |
|  | *Vittaforma corneum* | Intestinal microsporidiosis, and eye infections |
| **Microalgae** | *Alexandrium catenella* | Paralytic shellfish poisoning (PSP) |
|  | *Alexandrium minutum* | Paralytic shellfish poisoning (PSP) |
|  | *Alexandrium sp.* | Paralytic shellfish poisoning (PSP) |
|  | *Amphidinium carterae* | Paralytic shellfish poisoning (PSP) |
|  | *Amphidinium spp.* | Paralytic shellfish poisoning (PSP) |
|  | *Coolia monotis* | Neurotoxin poisoning |
|  | *Dinophysis acuminata* | Diarrheal shellfish poisoning (DSP) |
|  | *Dinophysis caudata* | Diarrheal shellfish poisoning (DSP) |
|  | *Dinophysis sp.* | Diarrheal shellfish poisoning (DSP) |
|  | *Gambierdiscus caribaeus* | Ciguatera |
|  | *Gambierdiscus toxicus* | Ciguatera |
|  | *Gymnodinium catenatum* | Paralytic shellfish poisoning (PSP) |
|  | *Karenia brevis* | Neurotoxic shellfish poisoning (NSP) |
|  | *Nitzschia longissima* | Amnesic shellfish poisoning (ASP) |
|  | *Nitzschia reversa* | Amnesic shellfish poisoning (ASP) |
|  | *Ostreopsis heptagona* | Neurotoxic shellfish poisoning (NSP) |
|  | *Prorocentrum concavum* | Diarrheal shellfish poisoning (DSP) |
|  | *Prorocentrum foraminosum* | Diarrheal shellfish poisoning (DSP) |
|  | *Prorocentrum hoffmanianum* | Diarrheal shellfish poisoning (DSP) |
|  | *Prorocentrum lima* | Diarrheal shellfish poisoning (DSP) |
|  | *Prorocentrum mexicanum* | Diarrheal shellfish poisoning (DSP) |
|  | *Prorocentrum minimum* | Paralytic shellfish poisoning (PSP) |
|  | *Prorocentrum rhathymum* | Diarrheal shellfish poisoning (DSP) |
|  | *Prorocentrum sculptile* | Diarrheal shellfish poisoning (DSP) |
|  | *Prorocentrum sipadanensis* | Diarrheal shellfish poisoning (DSP) |
|  | *Prorocentrum spp.* | Diarrheal shellfish poisoning (DSP) |
|  | *Pseudonitzschia calliantha* | Amnesic shellfish poisoning (ASP) |
|  | *Pseudonitzschia cuspidata* | Amnesic shellfish poisoning (ASP) |
|  | *Pseudonitzschia pungens* | Amnesic shellfish poisoning (ASP) |
|  | *Pseudonizschia delicatissima* | Amnesic shellfish poisoning (ASP) |
|  | *Pyrodinium bahamense* | Paralytic shellfish poisoning (PSP) |
| **Animalia; Nematoda: Roundworms** | *Ancylostoma spp.* | Cutaneous larva migrans |
|  | *Anisakis simplex* | Anisakiasis |
|  | *Ascaris lumbricoides* | Ascariasis |
|  | *Dracunculus medinensis* | Dracunculiasis |
|  | *Enterobius vermicularis* | Enterobiasis |
|  | *Eustrongylides sp.* | Eustrongyloidiasis |
|  | *Onchocerca volvulus* | Onchocerciasis |
|  | *Trichuris trichiura* | Trichuriasis |
| **Animalia; Platyhelminthes: Flatworms** | *Diphyllobothrium spp.* | Diphyllobothriosis |
|  | *Fasciola gigantica* | Fascioliasis |
|  | *Fasciola hepatica* | Fascioliasis |
|  | *Nanophyetus salminicola* | Nanophyetiasis |
|  | *Schistosoma spp.* | Schistosomiasis |
|  | *Taenia saginata* | Cysticercosis, taeniasis |
|  | *Taenia solium* | Cysticercosis, taeniasis |
|  | *Taenia sp.* | Cysticercosis, taeniasis |
| **Protozoa** | *Acanthamoeba spp.* | Keratitis, encephalitis |
|  | *Balantidium coli* | Balantidiasis |
|  | *Cryptosporidium parvum* | Stomach flu |
|  | *Cyclospora cayetanensis* | Cyclosporiasis |
|  | *Cystoisospora belli* | Isosporiasis |
|  | *Entamoeba histolytica* | Amoebiasis |
|  | *Giardia intestinalis* | Giardiasis |
|  | *Giardia lamblia* | Giardiasis |
|  | *Naegleria fowleri* | Meningoencephalitis |
|  | *Toxoplasma gondii* | Toxoplasmosis |
|  | *Trypanosoma cruzi* | Chagas |
| **Virus** | *Alphavirus Chikungunya virus* | Chikungunya fever |
|  | *Alphavirus sp.* | Encephalitis |
|  | *Arenavirus Junin virus* | Hemorrhagic fever |
|  | *Coronavirus spp.* | Pneumonia |
|  | *Ebolavirus spp.* | Ebola |
|  | *Enteovirus coxsackievirus* | Febrile illness, meningitis |
|  | *Enterovirus Human echovirus 30* | Meningitis |
|  | *Erythrovirus Human parvovirus* | Erythema |
|  | *Filovirus** | Hemorrhagic fever, joint pain |
|  | *Flavivirus spp.* | Yellow fever (sylvatic), Dengue, Zika |
|  | *Hantavirus* | Cardiopulmonary syndrome |
|  | *Hepacivirus Hepatitis C virus* | Liver inflammation |
|  | *Hepatovirus Hepatitis A virus* | Gastroenteritis |
|  | *Hepevirus Hepatitis E virus* | Viral infection of the liver |
|  | *Human herpesvirus 3* | Chickenpox, shingles |
|  | *Human herpesvirus spp.* | Genital herpes, Kaposi's sarcoma |
|  | *Human papillomavirus* | Epithelial tissue lesions |
|  | *Influenzavirus* | Influenza type A and B |
|  | *Lentivirus Human inmmunodeficiency virus 1* | Acquired Immune Deficiency Syndrome (AIDS) |
|  | *Lyssavirus rabies virus* | Rabies |
|  | *Mamastrovirus Human astrovirus* | Gastroenteritis |
|  | *Marbugvirus* | Hemorrhagic fever |
|  | *Mastadenovirus Human adenovirus* | Gastroenteritis, pharyngitis, conjunctivitis and urethritis |
|  | *Morbillivirus measles virus* | Measles |
|  | *Norovirus spp.* | Epidemic gastroenteritis |
|  | *Orthohepadnavirus Hepatitis B virus* | Viral hepatitis |
|  | *Orthopoxvirus Variola major* | Smallpox |
|  | *Orthoreovirus Mammalian orthoreovirus 3* | Gastroenteritis |
|  | *Poliovirus* | Poliomyelitis |
|  | *Polyomavirus JC polyomavirus* | Progressive multifocal leukoencephalopathy |
|  | *Respirovirus Human Parainfluenza virus 〤* | Obstructive laryngitis |
|  | *Respirovirus Human Parainfluenza virus 3* | Bronchiolitis and pneumonia |
|  | *Rhinovirus Human Rhinovirus* | Common cold |
|  | *Rotavirus Human rotavirus* | Gastroenteritis |
|  | *Rubivirus Rubella virus* | Rubella |
|  | *Rubulavirus Human Parainfluenza virus 2* | Obstructive laryngitis |
|  | *Rubulavirus Human Parainfluenza virus 4* | Upper and lower respiratory infections |
|  | *Rubulavirus sp.* | Mumps |
|  | *Sapovirus* | Gastroenteritis |
|  | *Togaviridae** | Arthritis, encephalitis, rubella |
| **Resistance genes** | *qnrA* | Quinolone resistance gene |
|  | *qnrB* | Quinolone resistance gene |
|  | *qnrS* | Quinolone resistance gene |
|  | *aac(6'Ib)* | Quinolone and aminoglycoside resistance gene |
|  | *qepA* | Fluoroquinolones resistance gene |
|  | *blaTEM, blaSHV, blaCTX-M* | Beta-lactam resistance genes |
|  | *aaDA* | Streptomycin-spectinomycin resistance genes |
|  | *mcr1* | Genes of resistance to imipenem, meropenem, piperacillin, ceftazidime, cefotaxime, ciprofloxacin and colistin |
|  | *qnrB4* | Genes of resistance to ceftazidime, cefotaxime, piperacillin, ciprofloxacin, imipenem, meropenem, and gentamicin |
|  | *blaIMP, blaVEB* | Genes of resistance to beta-lactams, imipenem, meropenem, and gentamicin |
|  | *blaOXA, bla_amp_C* | Genes of resistance to the newer generation of cephalosporins and carbapenems |
